# Divergent Regulation of Decidual Oxidative-Stress Response by NRF2 and KEAP1 in Preeclampsia with and without Fetal Growth Restriction

**DOI:** 10.3390/ijms23041966

**Published:** 2022-02-10

**Authors:** Siv Boon Mundal, Johanne Johnsen Rakner, Gabriela Brettas Silva, Lobke Marijn Gierman, Marie Austdal, Purusotam Basnet, Mattijs Elschot, Siril Skaret Bakke, Jenny Ostrop, Liv Cecilie Vestrheim Thomsen, Eric Keith Moses, Ganesh Acharya, Line Bjørge, Ann-Charlotte Iversen

**Affiliations:** 1 Centre of Molecular Inflammation Research (CEMIR), Department of Clinical and Molecular Medicine, Norwegian University of Science and Technology (NTNU), 7491 Trondheim, Norway; siv.boon@gmail.com (S.B.M.); johanne.j.rakner@ntnu.no (J.J.R.); gabrielabrettas@gmail.com (G.B.S.); lobke.gierman@ntnu.no (L.M.G.); marie.austdal@sus.no (M.A.); siril.s.bakke@ntnu.no (S.S.B.); jenny.ostrop@uib.no (J.O.); 2 Women’s Health and Perinatology Research Group, Department of Clinical Medicine, UiT—The Arctic University of Norway, 9037 Tromsø, Norway; purusotam.basnet@uit.no (P.B.); ganesh.acharya@ki.se (G.A.); 3 Department of Gynecology and Obstetrics, St. Olavs Hospital, Trondheim University Hospital, 7030 Trondheim, Norway; 4 Department of Research, Stavanger University Hospital, 4068 Stavanger, Norway; 5 Department of Obstetrics and Gynecology, University Hospital of Northern Norway, 9037 Tromsø, Norway; 6 Department of Circulation and Medical Imaging, Norwegian University of Science and Technology (NTNU), 7491 Trondheim, Norway; mattijs.elschot@ntnu.no; 7 Department of Radiology and Nuclear Medicine, St. Olavs Hospital, Trondheim University Hospital, 7030 Trondheim, Norway; 8 Department of Gynecology and Obstetrics, Haukeland University Hospital, 5058 Bergen, Norway; liv.vestrheim@uib.no (L.C.V.T.); line.bjorge@uib.no (L.B.); 9 Centre for Cancer Biomarkers CCBIO, Department of Clinical Science, University of Bergen, 5021 Bergen, Norway; 10 Menzies Institute for Medical Research, University of Tasmania, Hobart, TAS 7000, Australia; eric.moses@utas.edu.au; 11 Department of Clinical Science, Division of Obstetrics and Gynecology, Intervention and Technology, Karolinska Institutet, 141 86 Stockholm, Sweden

**Keywords:** antioxidant capacity, decidua, fetal growth restriction, KEAP1, NRF2, oxidative stress, preeclampsia, trophoblast

## Abstract

Utero-placental development in pregnancy depends on direct maternal–fetal interaction in the uterine wall decidua. Abnormal uterine vascular remodeling preceding placental oxidative stress and placental dysfunction are associated with preeclampsia and fetal growth restriction (FGR). Oxidative stress is counteracted by antioxidants and oxidative repair mechanisms regulated by the transcription factor nuclear factor erythroid 2-related factor 2 (NRF2). We aimed to determine the decidual regulation of the oxidative-stress response by NRF2 and its negative regulator Kelch-like ECH-associated protein 1 (KEAP1) in normal pregnancies and preeclamptic pregnancies with and without FGR. Decidual tissue from 145 pregnancies at delivery was assessed for oxidative stress, non-enzymatic antioxidant capacity, cellular NRF2- and KEAP1-protein expression, and NRF2-regulated transcriptional activation. Preeclampsia combined with FGR was associated with an increased oxidative-stress level and NRF2-regulated gene expression in the decidua, while decidual NRF2- and KEAP1-protein expression was unaffected. Although preeclampsia with normal fetal growth also showed increased decidual oxidative stress, NRF2-regulated gene expression was reduced, and KEAP1-protein expression was increased in areas of high trophoblast density. The trophoblast-dependent KEAP1-protein expression in preeclampsia with normal fetal growth indicates control of decidual oxidative stress by maternal–fetal interaction and underscores the importance of discriminating between preeclampsia with and without FGR.

## 1. Introduction

Preeclampsia is an inflammatory multisystem syndrome affecting 2–5% of pregnancies, and it is often further complicated by fetal growth restriction (FGR) [1,2,3]. Both disorders originate from placental dysfunction due to inadequate blood supply via decidual spiral arteries to the placenta. The decidua basalis develops from the endometrium at the blastocyst implantation site and is composed of spiral arteries, decidualized endometrial cells, maternal immune cells, fetal extravillous trophoblasts, fibrinoid layers and endometrial glands [4]. Close interactions between fetal extravillous trophoblasts and maternal cells are crucial for optimal remodeling of the decidual spiral arteries during pregnancy. Impaired spiral-artery remodeling causes malperfusion of the placenta and leads to placental oxidative-, endoplasmic reticulum (ER)- and inflammatory-stress culminating in established placental dysfunction. The dysfunctional placenta releases stress signals and anti-angiogenic factors to the maternal circulation, leading to endothelial dysfunction and, eventually, to the clinical signs of preeclampsia [1,2].

Reactive oxygen species (ROS) are constantly generated within cells as metabolic by-products, and low-to-moderate levels of ROS are physiological [5,6]. When the production of ROS overwhelms the tissue antioxidant defenses, oxidative stress occurs and causes cellular damage. ROS may provoke damage to multiple cellular organelles and ultimately be detrimental [7]. Nuclear factor erythroid 2-related factor 2 (NRF2) is the master regulator of cellular oxidative-stress responses and initiates the transcription of antioxidants and protective genes for oxidative repair and detoxification [8,9,10]. During oxidative stress, NRF2 is phosphorylated and translocates to the nucleus [11], binds the antioxidant response element (ARE), and initiates the transcription of antioxidants and protective genes [9]. The NRF2-binding protein Kelch-like ECH-associated protein 1 (KEAP1) is a negative regulator of NRF2 that ensures homeostasis by turning off the NRF2 transcriptional activity when not required, through a continuous degradation of the NRF2 protein [9]. KEAP1 inhibits NRF2 both by binding cytosolic NRF2 and preventing its translocation to the nucleus, and by removing nuclear NRF2 from the ARE [12,13,14]. In the case of oxidative stress, NRF2-activating electrophilic molecules may modify cysteine residues in KEAP1 and impair its function [15] or other mechanisms may disrupt KEAP1–NRF2 protein–protein interactions [16].

Although decidual oxidative stress plays an essential role in the establishment of normal placental function [5,17], excessive oxidative stress in the placenta, decidua and maternal circulation is central to the development of preeclampsia and FGR [5,18,19,20]. Placental NRF2 expression has been shown to be both upregulated [21,22] and downregulated [23] in preeclampsia. Decidual NRF2 is expressed by extravillous trophoblasts and maternal cells such as decidual stroma cells, myometrial cells and leukocytes, and is upregulated in preeclampsia [24,25]. The NRF2 expression and cellular distribution have not been determined in the decidua for different preeclampsia subgroups.

We previously performed decidual transcriptional profiling where the “NRF2-mediated oxidative stress response pathway” was shown to be dysregulated in preeclampsia [26]. A more comprehensive assessment of the decidual oxidative-stress response at the protein level is needed in order to improve our understanding of how it affects the maternal–fetal interaction and pregnancy outcomes. We aimed to determine the role of NRF2- and KEAP1-regulated oxidative-stress responses in decidual maternal and fetal cells in normal pregnancies and preeclamptic pregnancies with and without FGR.

## 2. Results

### 2.1. Characteristics of the Study Population

A total of 145 pregnant women were recruited to the study. As expected, preeclamptic pregnancies, both with and without FGR, were associated with elevated blood pressure, lower gestational age at delivery, reduced birth weight and placental weight, and increased occurrence in first pregnancies (Table 1). Preeclamptic pregnancies complicated by FGR had lower birth weight and placental weight, placental weight ratio, and gestational age at delivery compared to preeclamptic pregnancies without FGR.

### 2.2. Non-Enzymatic Antioxidant Capacity and Oxidative-Stress Levels in the Decidua

#### 2.2.1. Non-Enzymatic Antioxidant Capacity in the Decidua

**Figure 1:**
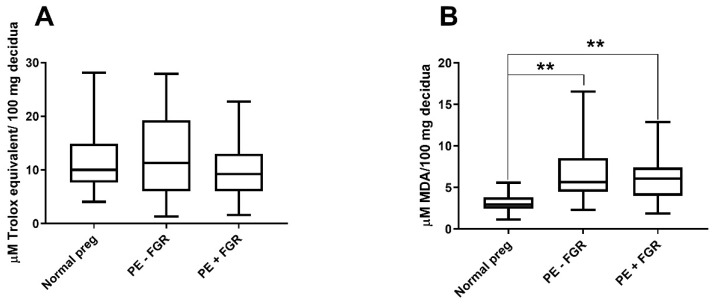
Non-enzymatic antioxidant capacity and oxidative-stress levels in decidua. Decidual non-enzymatic antioxidant capacity expressed as µM Trolox equivalent levels per 100 mg decidual tissue (**A**). Decidual oxidative-stress levels assessed by malondialdehyde (MDA) concentrations per 100 mg decidual tissue (**B**). Comparisons between normal pregnancies (preg) and preeclampsia (PE) with or without fetal growth restriction (FGR) (**A**,**B**). Boxes with medians extend from the 25th to 75th percentiles. ** *p* < 0.001.

Frozen decidual samples were available from 126 of the 145 pregnancies. Ten of these were excluded due to insufficient amounts or poor tissue quality, and one was excluded as an outlier. No differences in decidual non-enzymatic antioxidant capacity between preeclampsia with or without FGR when compared to normal pregnancies were observed (Figure 1A).

#### 2.2.2. Decidual Oxidative-Stress Levels

Of 126 available frozen decidual samples, 15 were excluded due to insufficient amount or poor tissue quality and one as an outlier. Significantly higher oxidative-stress levels were detected in preeclampsia both with and without FGR compared to normal pregnancies (*p* < 0.001) (Figure 1B).

### 2.3. NRF2-Regulated Transcriptional Activation

Eighty-six of the 145 included pregnancies had been analyzed in a previous decidual microarray transcriptional data set [26] and were reanalyzed for the current study. The transcriptional data were available for 51 normal pregnancies, 11 preeclamptic pregnancies without FGR, and 24 preeclamptic pregnancies with FGR. The comparison between preeclampsia with and without FGR showed that preeclampsia without FGR was associated with a reduced overall expression of NRF2-regulated transcripts (*p* = 0.048) and the transcripts for “antioxidant proteins” (*p* = 0.02) and the “chaperone and stress response proteins” (*p* < 0.05) (Table 2). In contrast, the comparison between preeclampsia with FGR and normal pregnancies showed that higher expression of transcripts for “chaperone and stress response proteins” was associated with preeclampsia with FGR (*p* < 0.001) (Table 2). The decidual expression of the main “antioxidant protein” heme oxygenase 1 (HO-1) (Supplementary Table S1) was significantly reduced in preeclampsia without FGR compared to preeclampsia with FGR (Table 2). The expression of the HO-1 protein was assessed by reanalyzing the data from a previous study [30]. HO-1 was expressed in most cell types in the decidua (Supplementary Figure S1), and the expression level was significantly higher in preeclampsia overall compared to normal pregnancies (*p* = 0.01) (Figure S2A). A comparison of the overall decidual HO-1 protein expression between preeclampsia with and without FGR showed no differences, but included only three cases of preeclampsia without FGR (Supplementary Figure S2B).

### 2.4. Cellular Quantitative Decidual NRF2 and KEAP1 Expression

**Figure 2:**
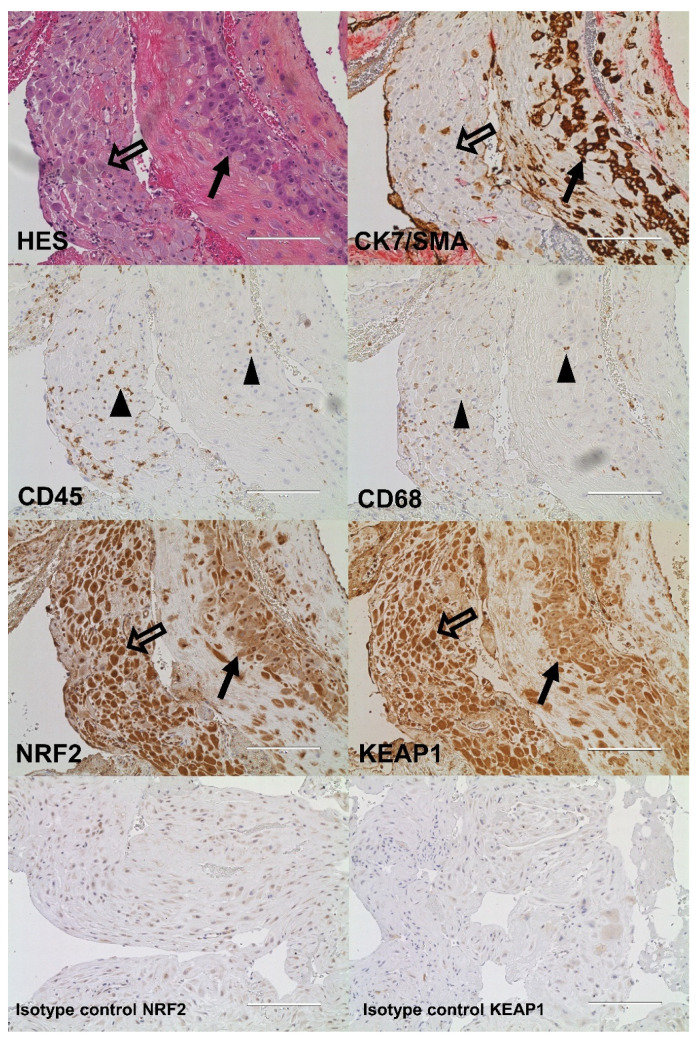
Decidual tissue from a preeclamptic pregnancy without fetal growth restriction stained as indicated with hematoxylin and eosin (HES) and markers for trophoblasts (cytokeratin 7 (CK7)), leukocytes (CD45), macrophages (CD68), nuclear factor erythroid 2-related factor 2 (NRF2), Kelch-like ECH-associated protein 1 (KEAP1), or negative isotype controls (20× magnification). CK7 was counterstained with smooth muscle actin (SMA). Black arrows indicate trophoblasts, transparent arrows indicate maternal decidual stroma cells, and black triangles indicate maternal leukocytes. Scale bar: 200 µM.

**Figure 3:**
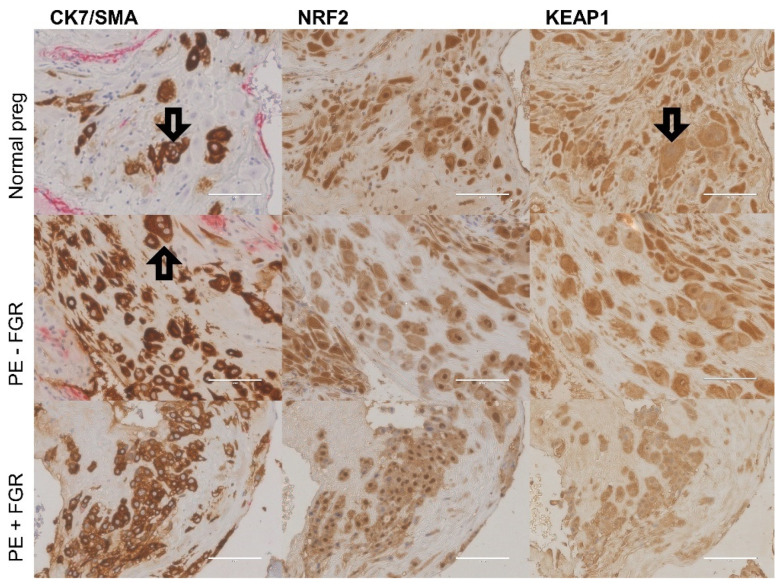
Decidual expression of trophoblasts (cytokeratin 7 (CK7)), nuclear factor erythroid 2-related factor 2 (NRF2), and Kelch-like ECH-associated protein 1 (KEAP1) in normal pregnancy, preeclampsia (PE) without fetal growth restriction (FGR), and PE with FGR (40× magnification). CK7 was counterstained with smooth muscle actin (SMA). The transparent arrows indicate examples of trophoblast giant cells. Scale bar: 100 µM.

The NRF2- and KEAP1-protein expression in the decidua was assessed in 88 and 82 pregnancies, respectively. In the decidua, extravillous trophoblasts were observed both clustered together surrounded by fibrinoid tissue and as single cells in close proximity to maternal decidual stroma cells, leukocytes, and macrophages (Figure 2). The interstitial clusters of extravillous trophoblasts contained defined multinucleated trophoblast giant cells (Figure 3).

The decidual expression of NRF2 and KEAP1 was detected in both fetal cells and in maternal tissue lacking trophoblasts (Figure 2). NRF2 and KEAP1 were strongly expressed by fetal extravillous trophoblasts, multinucleated trophoblast giant cells, maternal decidual stroma cells and leukocytes, while muscle cells/myometrial cells showed a weaker expression (Figure 2). The decidual NRF2 and KEAP1 expression was localized to the nucleus and cytoplasm and comparably distributed in normal and preeclamptic pregnancies (Figure 3). The nuclear localization of NRF2 was confirmed by the staining of the phosphorylated form of NRF2 (Supplementary Figure S3). The amount and density of decidual trophoblasts did not differ between normal and preeclamptic pregnancies (data not shown).

**Figure 4:**
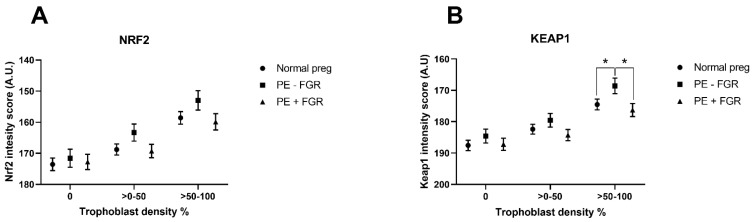
Decidual protein expression levels of nuclear factor erythroid 2-related factor 2 (NRF2) and Kelch-like ECH-associated protein 1 (KEAP1) in maternal cells and fetal trophoblasts, and maternal tissue without trophoblasts (0% trophoblast density). The expression of NRF2 (**A**) and KEAP1 (**B**) related to trophoblast density was compared between normal pregnancies (preg) and preeclampsia (PE) with or without fetal growth restriction (FGR). Expression levels are shown as estimated means with standard errors of means. * *p* < 0.05.

**Figure 5:**
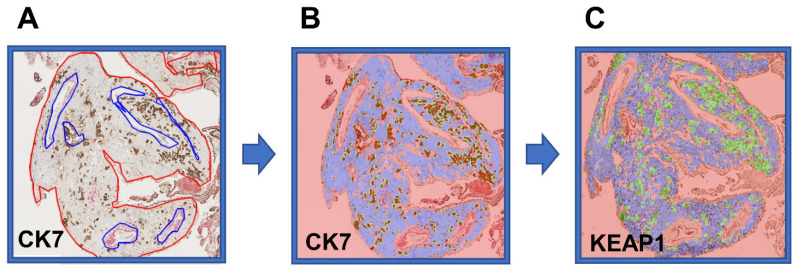
Automated identification of trophoblasts and maternal tissue in decidua for cellular quantification of nuclear factor erythroid 2-related factor 2 (NRF2)- and Kelch-like ECH-associated protein 1 (KEAP1)-protein expression using a custom MATLAB script. Tissue scans of slides stained with the trophoblast marker CK7 were used to determine included (red) and excluded (blue) tissue areas (**A**). Positive CK7 staining identified trophoblast-rich areas (green patches), and lack of CK7 identified maternal-tissue areas (blue patches) (**B**). A mask of patches was created for each decidua based on CK7-positive staining and used to relate NRF2- and KEAP1-expression levels to trophoblasts and maternal tissue in the spatially aligned NRF2 or KEAP1 tissue scans (**C**).

The decidual NRF2 and KEAP1 expression was quantified in decidual trophoblasts and in maternal tissue without trophoblasts (Figure 4 and Supplementary Table S2), using a novel automated protein-quantification method (Figure 5). The trophoblast-dependent NRF2 expression tended to be higher in preeclamptic pregnancies without FGR than in normal pregnancies and preeclamptic pregnancies with FGR (*p* = 0.10 and *p* = 0.09, respectively) (Figure 4A and Supplementary Table S2). No difference in NRF2-protein expression was detected in maternal tissue without the presence of trophoblasts (Figure 4A). The decidual KEAP1-expression pattern differed from the pattern for NRF2 by being more strongly located to extravillous trophoblast-rich areas, and decidual KEAP1 in trophoblast-rich areas showed increased expression in preeclampsia without FGR compared to both normal pregnancies and preeclampsia with FGR (*p* = 0.049 and *p* = 0.02, respectively) (Figure 4B). The KEAP1-protein expression in maternal tissue (Figure 4B) and the overall KEAP1 expression (protein and mRNA) in decidual tissue did not differ between groups (data not shown).

## 3. Discussion

This study shows the protein expression of the oxidative-stress regulator NRF2 and its inhibitor KEAP1 by both extravillous trophoblasts and maternal cells in the decidua. By using a novel automated image-based quantification of protein expression, we identified a distinct decidual NRF2-regulated oxidative-stress response in preeclampsia with normal fetal growth. This response was characterized by increased trophoblast-dependent KEAP1 expression and corresponding inhibition of the expression of NRF2-regulated antioxidant-response genes, and with a further corresponding increase in the decidual oxidative-stress levels. Preeclamptic pregnancies with FGR showed increased NRF2-regulated stress-response genes and oxidative stress, but a maternal or fetal effect on decidual NRF2- or KEAP1-protein expression was not identified in those pregnancies.

Our findings are in accordance with other studies that have shown decidual NRF2 expression in extravillous trophoblasts and maternal cells such as decidual stroma cells, myometrial cells, and leukocytes [24,25]. However, while Kweider et al. described exclusive cytoplasmic expression of NRF2 in extravillous trophoblasts [24], our findings show a clear nuclear expression of NRF2 in several cell types, paralleled by transcriptional functionality. This discrepancy may be due to the use of different antibodies. In smaller study populations, the decidual expression of NRF2 has been shown to be upregulated in trophoblasts in early-onset preeclampsia with FGR [24] and downregulated in isolated FGR [25]. To our knowledge, decidual KEAP1-protein expression has not previously been reported, and the presented cytoplasmic and nuclear expression of KEAP1 is supported by its role in sequestering cytoplasmic NRF2 through an active Crm1/exportin-dependent nuclear-transport mechanism [32]. We identified FGR-associated regulation of the oxidative-stress response in preeclampsia, as evidenced by increased decidual trophoblast-dependent KEAP1-protein expression in preeclampsia without FGR. Similar upregulation of Keap1 mRNA could not be detected by microarray, probably since it was performed on decidual tissue consisting of both maternal and fetal cells.

A microarray meta-analysis of the placenta has shown transcriptional downregulation of Nrf2 and Keap1 in preeclampsia [33]. Both reduced [23] and increased [21,22] placental nuclear NRF2-protein expression have been reported in preeclampsia. Likewise, studies assessing NRF2-regulated enzyme activity and the protein expression of NRF2 targets have reported both increases and reductions associated with preeclampsia [25,34,35,36,37]. Our novel identification of a diverging regulation of NRF2-regulated oxidative-stress responses in preeclampsia subgroups provides a probable explanation for the lack of consistency in existing data that have not distinguished between preeclampsia subgroups. The NRF2-regulated genes were assessed as overall response pathways in this study. Further gene or protein expression analysis of specific NRF2 target genes would strengthen the findings. Newer technologies such as single-cell RNA sequencing may provide a better approach to understanding this heterogenous tissue consisting of both maternal and fetal cells [38,39].

The increased NRF2-mediated gene activation in preeclampsia with FGR presented here was not supported by the decidual transcriptomics profiling by Tong et al., but they included other subgroups of preeclampsia (i.e., early onset and late onset) and had only three individuals per group [38,40]. The distinct NRF2-mediated regulation correlates, however, with our previous findings of increased decidual ER-stress responses associated with preeclampsia with FGR [41]. Oxidative and ER stress are closely linked and activate NRF2 [42]. ER stress results in the accumulation of misfolded proteins. The increased NRF2-regulated “chaperone and stress response proteins” genes in preeclampsia with FGR may indicate that the accumulation of unfolded proteins in the decidua is a central challenge in this preeclampsia subgroup. The downstream effect of placental ER stress and responses is implicated in FGR, as it may lead to perturbations of post-translational modifications and reduced translation of proteins, resulting in a small and insufficient placenta [42].

The decidual expression of KEAP1 was selectively increased in preeclampsia without FGR, potentially resulting in a reduced NRF2/KEAP1 ratio, supporting a net inhibition of NRF2 activation as an explanation for the corresponding reduction in the NRF2-regulated gene expression. This KEAP1 inhibition by fetal cells was associated with improved fetal outcomes, suggesting that decidual KEAP1 represents a fetal protective mechanism in preeclampsia; this is a novel observation in humans. Our findings are partly supported in a study of mice with pregnancy-associated hypertension where KEAP1 knockdown resulted in reduced fetal weight, and reduced placental angiogenesis stimulated by increased ROS production was suggested as a causative mechanism [43]. In further agreement, NRF2 knockdown has been shown to improve maternal and fetal outcomes in similar murine studies [44,45]. Accepting somewhat higher levels of oxidative stress to improve the placental vascularization in pregnancies with vascular dysfunction, such as in preeclampsia, may be a beneficial fetal compromise. Stronger KEAP1 inhibition may allow for a moderate increase in ROS, stimulating beneficial and immune-suppressive T-cell activity in the decidual microenvironment [6,46].

Chronic NRF2 activation may affect the renin–angiotensin system [47,48,49]. A prolonged activation of NRF2 has been related to hypertension, kidney injury, and cardiac maladaptation, and these adverse effects were reverted by NRF2 inhibition in murine diabetes [48] and cardiac-pressure-overload models [47]. Prolonged NRF2 activation results in an increased angiotensin II/angiotensin 1-7 ratio, facilitating development of hypertension [48]. The same perturbation of this ratio is observed in maternal plasma and urine in preeclampsia [50], and may be linked to chronic NRF2 activation from oxidative stress. Increased decidual expression of angiotensin II has been reported in preeclampsia [51]. The decidual angiotensin II could act on adjacent fetal chorionic villi, where angiotensin II receptor type 1 expression is increased in preeclampsia, and thereby induce vasoconstriction that impairs fetal blood supply [51,52,53]. Our study supports the implications of these previous studies, that chronic stress-induced decidual NRF2 activation might be a detrimental step in the development of preeclampsia potentially resulting in FGR through its effect on the renin–angiotensin system. Increased KEAP1 expression in preeclampsia without FGR, as observed here, potentially counteracts these adverse effects of NRF2 activation.

This study substantiates a divergent regulation of the decidual NRF2-mediated oxidative-stress response in preeclampsia with and without FGR, and suggests that the inhibitor KEAP1 is an important regulator. The complex role of NRF2-regulated oxidative stress in pregnancy warrants a more detailed characterization of the NRF2 system locally at the maternal–fetal interface throughout gestation, in relation to maternal–fetal outcomes. The unbiased automated cellular quantification method developed for our study allows for such follow-up studies. The trophoblast-dependent downregulation of NRF2-mediated oxidative-stress responses identifies a role for decidual maternal–fetal interaction in the regulation of oxidative-stress responses in pregnancy.

## 4. Materials and Methods

### 4.1. Study Participants and Decidual Biopsies

The Preeclampsia Study includes healthy and preeclamptic singleton pregnancies delivered by caesarean section (CS) in the absence of labor at St. Olavs and Haukeland University Hospitals between 2002 and 2012. Pregnant women diagnosed with preeclampsia with or without FGR were included as cases. Healthy normotensive pregnant women with no previous history of preeclampsia or FGR were included as normal pregnant controls. For the current study, women with immunosuppressive medications, pre-existing hypertension, or gestational diabetes mellitus were excluded. Preeclampsia was defined as persistent hypertension exceeding 140/90 mmHg plus proteinuria ≥0.3 g/24 h or ≥+1 by dipstick after 20 weeks of gestation. FGR was diagnosed by serial ultrasound measurements showing reduced intrauterine growth, or by birth weights < the 5th percentile of Norwegian reference curves [54] combined with clinically and sonographically suspected FGR and/or postpartum defined placental pathology.

Decidua basalis tissue was collected by vacuum suction of the placental bed during caesarean section [18,55]. None of the women showed signs of labor prior to the caesarean section. The samples were placed in RNAlater or in 10% neutral-buffered formalin and paraffin embedded or snap frozen in liquid nitrogen within 30 min of collection.

The Norwegian Regional Committee for Medical and Health Research Ethics approved the study (REC no. 2012/1040), and written informed consent was obtained from each participant.

### 4.2. Non-Enzymatic Antioxidant-Capacity Assay

The non-enzymatic antioxidant capacity was measured as the 3-ethylbenzothiazoline-6-sulphonic acid (ABTS)-radical-scavenging activity [56]. In brief, ABTS radicals were generated by mixing 2 mL each of ABTS (7.4 mM, #MAK187, Sigma-Aldrich, St. Louis, MO, USA, Total Antioxidant Assay KIT) and potassium peroxodisulfate (2.6 mM, #60489, Sigma-Aldrich, St. Louis, MO, USA). Decidual tissue lysates were prepared at 4 °C by homogenizing tissue samples in assay buffer from the Total Antioxidant Assay KIT (#MAK187, Sigma-Aldrich, St. Louis, MO, USA) (50 mg/250 µL) with a probe sonicator (10 s with 4 cycles/s) and centrifuged (12,000× *g* for 15 min) before collecting the supernatant. Reactions were carried out by incubating 190 μL of ABTS-radical solution and 10 μL of decidual lysate for 30 min. The green color of the ABTS radicals scavenged by decidual-lysate antioxidants was measured spectrophotometrically at 731 nm. The vitamin E equivalent Trolox (Sigma-Aldrich, St. Louis, MO, USA, Total Antioxidant Assay KIT) was used as a standard and quantified as μM Trolox/100 mg of decidua, representing the non-enzymatic antioxidant capacity.

### 4.3. Measuring Oxidative-Stress Levels by a Malondialdehyde (MDA) Assay

The total decidual MDA content analyzed with the Lipid Peroxidation (MDA) Assay Kit (#MAK085, Sigma-Aldrich, St. Louis, MO, USA) was used as a measurement of the decidual oxidative-stress levels [57]. In brief, a mixture of 100 µL of decidual extracts (10 µg of tissue/300 µL of extracting buffer) and 300 µL of thiobarbituric acid (TBA, #MAK085, Sigma-Aldrich Assay Kit, St. Louis, MO, USA) solution was incubated at 95 °C for 60 min. Of the reaction mixture, 150 µL was analyzed spectrophotometrically in duplicate at 532 nm. The decidual MDA level was estimated using the MDA standard provided with the kit.

### 4.4. NRF2-Regulated Transcriptional Activation

Decidual microarray transcriptional data from the pregnancies included in this study were published previously [26], and were preprocessed in Sequential Oligogenic Linkage Analysis Routines (SOLAR) [26,58], in accordance with the Minimum Information About a Microarray Experiment (MIAME) guidelines [59]. The data were submitted to ArrayExpress (www.ebi.ac.uk/arrayexpress/ (accessed on 4 May 2014)) under accession no. E-TABM-682. For the current study, downstream targets of NRF2 in “the NRF2-mediated oxidative-stress response pathway” were divided into five functional gene sets identified by Ingenuity Pathway Analysis (QIAGEN Inc., Germantown, MD, USA); 1, “antioxidant proteins”; 2, “phase I and II metabolizing enzymes”; 3, “chaperone and stress response proteins”; 4, “phase III detoxifying proteins”; and 5, “ubiquitination and proteasomal degradation” (Supplementary Table S1). Gene-set enrichment analysis (100 permutations) was run on the five gene sets in the Partek Genomics Suite 6.6 [31].

### 4.5. Immunohistochemistry

Parallel decidual tissue sections (3 μm) were pre-treated in PT link (#PT101, Dako, Glostrup, Denmark) using target retrieval solution (#K8004, Dako, Glostrup, Denmark) at 97 °C for 20 min, and next treated with peroxidase blocking solution (#K4007 or #K5361, Dako, Glostrup, Denmark). The tissue sections were incubated with primary antibodies for KEAP1 (1:150, #10503-2-AP, Proteintech, Rosemount, IL, USA, room temperature for 40 min); NRF2 (1:200, #PA1828, Bosterbio, Pleasanton, CA, USA, overnight at 4 °C); pNRF2 (1:300, #ab76026, Abcam, Cambridge, UK, room temperature for 40 min); cytokeratin 7 (CK7) (1:300, #M7018, Dako, Glostrup, Denmark, room temperature for 45 min); CD45 (1:150, #M0701, Dako, Glostrup, Denmark, room temperature for 40 min); or CD68 (1:6000, #M0718, Dako, Glostrup, Denmark, room temperature for 40 min). All the sections were incubated for 30 min with HRP-labeled polymer (#K4007, Dako, Glostrup, Denmark) and for 10 min with DAB+ as a chromogen (1:50, #K4007 or #K5361, Dako, Glostrup, Denmark). The CK7 sections were double-stained with smooth muscle actin antibodies (1:300, #M0851, Dako, Glostrup, Denmark) with the EnVision G|2 Doublestain System Rabbit/Mouse (DAB+/Permanent Red) Kit system (#K5361, Dako, Glostrup, Denmark). The staining was performed using an Autostainer Plus (#S3800, Dako, Glostrup, Denmark) for KEAP1 and CK7, and manually for NRF2. The sections were counterstained with hematoxylin. Negative isotype controls for KEAP1 and NRF2 were included (1:67, Rabbit IgG #NBP2-24891, Novus, St. Charles, MO, USA, and 1:240, CD3 #A0452, Dako, Glostrup, Denmark). Additional routine staining with hematoxylin (75290, Chemi-Teknik, Oslo, Norway), erythrosine 239 (720-0179, VWR, Radnor, PA, USA), and saffron (75100, Chemi-Teknik, Oslo, Norway) (HES) was performed using a Sakura Tissue-Tek © Prisma Stainer^TM^ (Sakura Finetek, Alphen aan den Rijn, the Netherlands). The reanalysis of HO-1 expression from a previous study [30] is included in the Supplementary Materials.

### 4.6. Automated Quantification of Protein Expression

Parallel sections of decidual tissue stained for NRF2, KEAP1, and CK7 by immunohistochemistry were used for protein quantification. Tissue scans were obtained with the EVOS^TM^ FL Auto Imaging System (Thermo Fisher Scientific, Waltham, MA, USA) using 20× magnification and defined microscope settings. To ensure a representative analysis, each tissue scan consisted of 9 to 100 bright-field TIFF images (2048 × 1536 pixels) per sample section depending on the amount of available tissue. A custom ImageJ script was used for the background correction and stitching of images [60,61,62]. The large tissue scans were further analyzed using custom MATLAB scripts (MathWorks, Natick, MA, USA, version 2017a) developed for the identification and automatic quantification of staining intensity [63,64,65], with the examiner blinded to the pregnancy outcome. Regions of the decidua with muscle cells, villous placental tissue, blood vessels, endometrial glands, and poor morphology were excluded by manually defining regions of disinterest (Figure 5A). A mask of patches (50 × 50 pixels, 662 µm × 662 µm) defining trophoblasts and maternal tissue without trophoblasts was created for each decidua based on CK7-positive staining (Figure 5B). The created masks were used to relate NRF2- and KEAP1-expression intensity to trophoblasts and maternal tissue in the spatially aligned NRF2 and KEAP1 tissue scans (Figure 5C). Trophoblasts were automatically counted, and the trophoblast density was calculated as the total number of trophoblasts divided by the total area of tissue (mm^2^). The average NRF2- and KEAP1-intensity values were grouped according to trophoblast density; as maternal tissue (0% trophoblasts), low trophoblast density (>0–50%), and high trophoblast density (>50%). The overall decidual KEAP1-expression intensity was calculated as the average value of all the positive patches. The decidual protein-expression-intensity values were measured as gray-level-intensity values ranging from 0 (the absence of color, black) to 255 (the presence of all colors, white) after conversion from RGB to grayscale images. The staining intensity is, therefore, inversely proportional to the protein-expression level.

### 4.7. Statistical Methods

The statistical analyses were performed using the SPSS v. 25, GraphPad Prism 7.03, and Partek Genomic Suite 6.6 software. For the clinical data, one-way ANOVA or Kruskal–Wallis tests with Tukey’s or Dunn’s test, respectively, were used for comparisons of continuous variables, while Fisher’s exact test was applied for categorical variables. For the non-enzymatic antioxidant capacity and oxidative-stress levels, outliers were detected with the Robust regression and Outlier removal (ROUT) method in GraphPad Prism 7.03, and the Kruskal–Wallis with Dunn’s test was used for comparisons.

The mRNA level of Keap1 from the decidual microarray data set was compared between normal pregnancies and pregnancies with preeclampsia with or without FGR by the Kruskal–Wallis test.

For the immunohistochemistry data, the amount and density of decidual trophoblasts were compared between the study groups by one-way ANOVA with Tukey’s test for pairwise comparisons. To compare the NRF2- and KEAP1-protein expression in maternal tissue (defined by 0% trophoblast density) and the overall decidual KEAP1-protein expression between the study groups, a linear regression model with the recruitment location and study group as additional covariates was used. To compare the NRF2 and KEAP1 expression in trophoblast-containing tissue (>0–50 or >50% trophoblasts), a linear mixed model with the recruitment location and trophoblast density interval as fixed-effects variables was used. Within-subject correlations were accounted for by including a subject-specific random intercept. The significance level was set to 0.05.

## Figures and Tables

**Table 1:** Clinical characteristics of included pregnancies.

	Normal Pregnancies (n = 70)	PE without FGR (n = 28)	PE with FGR (n = 47)
Baseline characteristics			
Maternal age, years	31.8 (±5.1)	30.5 (±4.9)	30.9 (±5.5)
BMI, kg/m^2^ *	25.2 (±4.2)	26.5 (±6.6)	27.2 (±5.5) ^†^
Primipara, n (%)	12 (17)	17 (61) ^‡^	26 (55) ^‡^
Characteristics at delivery			
Systolic BP, mmHg ^§^	121.1 (±12.8)	165.1 (±21.2) ^‡^	161.6 (±19.8) ^‡^
Diastolic BP, mmHg ^§^	73.4 (±8.6)	102.0 (±12.4) ^‡^	99.6 (±8.9) ^‡^
Gestational age, weeks	38.5 (±0.9)	33.1 (±3.9) ^‡^	30.1 (±3) ^‡#^
Severe PE ^||^, n (%)	n.a.	23 (82)	34 (72)
Early onset PE (<34 weeks), n (%)	n.a.	15 (54)	40 (85) ^†^
Placental weight, g	620 (193)	450 (238) ^‡^	275 (140) ^‡#^
Fetal birth weight, g	3621 (±474)	2208 (±955) ^‡^	1182 (±456) ^‡^
Placental weight ratio **	1.04 (0.35)	0.90 (0.35) ^‡^	0.60 (0.27) ^‡#^

BMI, body mass index; BP, blood pressure; FGR, fetal growth restriction, n.a., not applicable; PE, preeclampsia. Continuous variables, listed as means (±standard deviations) or medians (interquartile ranges), were assessed for differences between groups by one-way ANOVA with Tukey’s post hoc test or Kruskal–Wallis with Dunn’s post hoc test. Categorical variables, listed as numbers (percentages in parentheses), were assessed for differences between groups by Fisher’s exact test. * Maternal BMI at first antenatal care visit (first trimester). Information is missing for three women (2%). † Significantly different when comparing PE without FGR and PE with FGR to normal pregnancies, *p* < 0.05. ‡ Significantly different when comparing PE without FGR and PE with FGR to normal pregnancies, *p* < 0.001. § BP from last antenatal care visit or before the cesarean section. Information is missing for four women (3%). # Significantly different when comparing PE with FGR to PE without FGR, *p* < 0.001. ^||^ PE was classified as severe if one or more severe clinical features were present [27,28]. ** Calculated as observed/expected placental weight according to gestational age and sex in a Norwegian normogram [29]. Information is missing from one woman (0.6%).

**Table 2:** Gene-set enrichment analysis of NRF2-regulated functional gene sets.

Comparison between Diagnostic Groups		Gene-Set Description	No. of Transcr.	ES	NES	*p*-Value
PE − FGR	Normal preg	Antioxidant proteins	18	−0.47	−1.39	0.08
PE − FGR	Normal preg	Phase I and II metabolizing enzymes	48	−0.25	−0.85	0.70
PE − FGR	Normal preg	Chaperone and stress response proteins	43	−0.23	−0.82	0.76
PE − FGR	Normal preg	Phase III detoxifying proteins	4	0.31	0.59	0.90
PE − FGR	Normal preg	Ubiquitination/proteasomal degradation	5	0.37	0.74	0.77
PE − FGR	Normal preg	All five gene sets	118	−0.24	−1.01	0.44
PE + FGR	Normal preg	Antioxidant proteins	18	0.46	1.38	0.10
PE + FGR	Normal preg	Phase I and II metabolizing enzymes	48	−0.33	−1.23	0.24
PE + FGR	Normal preg	Chaperone and stress response proteins	43	0.45	1.73	<0.001
PE + FGR	Normal preg	Phase III detoxifying proteins	4	0.55	1.07	0.45
PE + FGR	Normal preg	Ubiquitination/proteasomal degradation	5	0.71	1.43	0.07
PE + FGR	Normal preg	All five gene sets	118	0.32	1.32	0.08
PE − FGR	PE + FGR	Antioxidant proteins	18	−0.60	−1.66	0.02
PE − FGR	PE + FGR	Phase I and II metabolizing enzymes	48	0.31	0.99	0.55
PE − FGR	PE + FGR	Chaperone and stress response proteins	43	−0.44	−1.59	<0.05
PE − FGR	PE + FGR	Phase III detoxifying proteins	4	−0.60	−1.06	0.45
PE − FGR	PE + FGR	Ubiquitination/proteasomal degradation	5	−0.71	−1.43	0.07
PE − FGR	PE + FGR	All five gene sets	118	−0.36	−1.41	<0.05

ES, enrichment score [31]; FGR, fetal growth restriction; NES, normalized enrichment score [31]; PE, preeclampsia; preg, pregnancy.

## Data Availability

The data presented in this study are available on request from the corresponding author. The data are not publicly available due to privacy.

## Supplementary Materials

Supporting information can be found at https://www.mdpi.com/article/10.3390/ijms23041966/s1.

## Abbreviations

ABTS	3-ethylbenzothiazoline-6-sulphonic acid
ARE	Antioxidant response element
CK7	Cytokeratin 7
ER	Endoplasmic reticulum
FGR	Fetal growth restriction
KEAP1	Kelch-like ECH-associated protein 1
MDA	Malondialdehyde
HO-1	Heme oxygenase 1
NRF2	Nuclear factor erythroid 2-related factor 2
ROS	Reactive oxygen species
