# Supplementary material for: Divergent Regulation of Decidual Oxidative-Stress Response by NRF2 and KEAP1 in Preeclampsia with and without Fetal Growth Restriction
Source: Int J Mol Sci. 2022 Feb 10;23(4):1966. doi: 10.3390/ijms23041966 (PMC8875334; doi:10.3390/ijms23041966)
Supplement: Supplementary file 1 [file ijms-23-01966-s001.zip › ijms-1515067-supplementary.pdf]

Supplementary Materials

Divergent Regulation of Decidual Oxidative-Stress Response by NRF2 and KEAP1 in Preeclampsia with and without Fetal Growth Restriction

Siv Boon Mundal [1,2], Johanne Johnsen Rakner [1], Gabriela Brettas Silva [1,3], Lobke Marijn Gierman [1,3], Marie Austdal [1,3,4] Purusotam Basnet [2,5], Mattijs Elschot [6,7], Siril Skaret Bakke [1], Jenny Ostrop [1], Liv Cecilie Vestrheim Thomsen [8,9], Eric Keith Moses [10], Ganesh Acharya [2,11], Line Bjørge [8,9] and Ann-Charlotte Iversen [1,3,*]

1. Centre of Molecular Inflammation Research (CEMIR), Department of Clinical and Molecular Medicine, Norwegian University of Science and Technology (NTNU), 7491 Trondheim, Norway; siv.boon@gmail.com (S.B.M.); johanne.j.rakner@ntnu.no (J.J.R.); gabrielabrettas@gmail.com (G.B.S.); lobke.gierman@ntnu.no (L.M.G.); marie.austdal@sus.no (M.A.); siril.s.bakke@ntnu.no (S.S.B.); jenny.ostrop@uib.no (J.O.); ann-charlotte.iversen@ntnu.no (A.-C.I.)
2. Women's Health and Perinatology Research Group, Department of Clinical Medicine, UiT-The Arctic University of Norway, 9037 Tromsø, Norway; purusotam.basnet@uit.no (P.B.); ganesh.acharya@ki.se (G.A.)
3. Department of Gynecology and Obstetrics, St. Olavs hospital, Trondheim University Hospital, 7030 Trondheim, Norway
4. Department of Research, Stavanger University Hospital, 4068 Stavanger, Norway
5. Department of Obstetrics and Gynecology, University Hospital of Northern Norway, 9037 Tromsø, Norway
6. Department of Circulation and Medical Imaging, NTNU, 7491 Trondheim, Norway; mattijs.elschot@ntnu.no
7. Department of Radiology and Nuclear Medicine, St. Olavs hospital, Trondheim University Hospital, 7030 Trondheim, Norway
8. Department of Gynecology and Obstetrics, Haukeland University Hospital, 5058 Bergen, Norway; liv.vestrheim@uib.no (L.C.V.T.); line.bjorge@uib.no (L.B.)
9. Centre for Cancer Biomarkers CCBIO, Department of Clinical Science, University of Bergen, 5021 Bergen, Norway
10. Menzies Institute for Medical Research, University of Tasmania, 7000 Hobart Tasmania, Australia;
eric.moses@utas.edu.au
11. Division of Obstetrics and Gynecology, Department of Clinical Science, Intervention and Technology,
Karolinska Institutet, 141 86 Stockholm, Sweden

* Correspondence: ann-charlotte.iversen@ntnu.no; Tel.:+47-93283877

Supplementary Materials and Methods

Expression and quantification of heme oxygenase (HO-1) in the decidua

We have reanalyzed a previous study of the stress response enzyme HO-1 in this cohort [1]. The HO-1 staining of decidual tissue sections by immunohistochemistry (dilution 1:800, #ab3470, Abcam, Cambridge, UK, room temperature for 40 minutes) in the previous work was evaluated manually, and we therefore performed a new automated quantification on the same decidual sections stained for HO-1 expression. Decidual areas with poor aberrant morphology were excluded by manually defining regions of disinterest. The images were analyzed by binary masks created by segmentation based on RGB color values using the color threshold app in MATLAB. The decidual HO-1 expression intensity was measured as the average intensity in a mask selecting only the tissue and not the background. The intensity values were measured as gray-level intensity values ranging from 0 (absence of color, black) to 255 (presence of all colors, white) after conversion from RGB to grayscale images. Staining intensity is therefore inversely proportional to the protein expression level. The decidual HO-1 protein expression was compared between normal pregnancies and pregnancies with preeclampsia by two-tailed unpaired t-test.

Table S1. NRF2 regulated transcripts in the "NRF2-mediated oxidative stress response"-pathway.

	**Antioxidant proteins**	
**Symbol**	**Entrez gene name**	**Illumina ID**
ATF4	activating transcription factor 4	ILMN_1672128
CAT	Catalase	ILMN_1805905
FTH1	ferritin heavy chain 1	ILMN_1683146
FTL	ferritin light chain	ILMN_1814823
GPX2	glutathione peroxidase 2	ILMN_1662776
GSR	glutathione-disulfide reductase	ILMN_1775182
HO-1	heme oxygenase 1	ILMN_1800512
MAFF	MAF bZIP transcription factor F	ILMN_1659955
MAFG	MAF bZIP transcription factor G	ILMN_1692260
MAFK	MAF bZIP transcription factor K	ILMN_1705986
NRF2	nuclear factor, erythroid 2 like 2	ILMN_1790909
PRDX1	peroxiredoxin 1	ILMN_1710159
SOD1	superoxide dismutase 1	ILMN_1662438
SOD2	superoxide dismutase 2	ILMN_1792922
SOD3	superoxide dismutase 3	ILMN_1690034
SQSTM1	sequestosome 1	ILMN_1703856
TRXR1	thioredoxin reductase 1	ILMN_1717056
TXN	Thioredoxin	ILMN_2038776
	**Phase I and II metabolizing enzymes**	
**Symbol**	**Entrez gene name**	**Illumina ID**
AKR	aldo-keto reductase family 1 member A1	ILMN_1774938
AKR1B1	aldo-keto reductase family 1 member B	ILMN_1701731
AKR1C3	aldo-keto reductase family 1 member C3	ILMN_1713124
AKR7A2	aldo-keto reductase family 7 member A2	ILMN_1677043
AKR7A3	aldo-keto reductase family 7 member A3	ILMN_1676592
AOX1	aldehyde oxidase 1	ILMN_1767113
CBR1	carbonyl reductase 1	ILMN_1809003

EPHX1	epoxide hydrolase 1	ILMN_1701025
FMO1	flavin containing monooxygenase 1	ILMN_1684401
GCLC	glutamate-cysteine ligase catalytic subunit	ILMN_1730575
GCLM	glutamate-cysteine ligase modifier subunit	ILMN_1788547
GSTA1	glutathione S-transferase alpha 1	ILMN_1701831
GSTA2	glutathione S-transferase alpha 2	ILMN_1655613
GSTA3	glutathione S-transferase alpha 3	ILMN_1769083
GSTA4	glutathione S-transferase alpha 4	ILMN_1771964
GSTA5	glutathione S-transferase alpha 5	ILMN_1788122
GSTK1	glutathione S-transferase kappa 1	ILMN_1725241
GSTM1	glutathione S-transferase mu 1	ILMN_1668134
GSTM2	glutathione S-transferase mu 2	ILMN_1713162
GSTM3	glutathione S-transferase mu 3	ILMN_1736184
GSTM4	glutathione S-transferase mu 4	ILMN_1651800
GSTM5	glutathione S-transferase mu 5	ILMN_1750790
GSTO1	glutathione S-transferase omega 1	ILMN_1808196
GSTO2	glutathione S-transferase omega 2	ILMN_1740234
GSTP1	glutathione S-transferase pi 1	ILMN_1679809
GSTT1	glutathione S-transferase theta 1	ILMN_1730054
GSTT2/GSTT2B	glutathione S-transferase theta 2 (gene/pseudogene)	ILMN_1738473
MGST1	microsomal glutathione S-transferase 1	ILMN_1694849
MGST2	microsomal glutathione S-transferase 2	ILMN_1802027
MGST3	microsomal glutathione S-transferase 3	ILMN_1751956
NQO1	NAD(P)H quinone dehydrogenase 1	ILMN_1720282
NQO2	N-ribosyldihydronicotinamide:quinone reductase 2	ILMN_1712918
UGT	solute carrier family 35 member A2	ILMN_1792135
CYP21A2	cytochrome P450 family 21 subfamily A member 2	ILMN_1773082
CYP2A6	cytochrome P450 family 2 subfamily A member 6	ILMN_1651636
CYP2C8	cytochrome P450 family 2 subfamily C member 8	ILMN_1769155
CYP2C9	cytochrome P450 family 2 subfamily C member 9	ILMN_1670652
CYP2D6	cytochrome P450 family 2 subfamily D member 6	ILMN_1740648
CYP2E1	cytochrome P450 family 2 subfamily E member 1	ILMN_1665437
CYP2J2	cytochrome P450 family 2 subfamily J member 2	ILMN_1758731
CYP2R1	cytochrome P450 family 2 subfamily R member 1	ILMN_1762498
CYP2S1	cytochrome P450 family 2 subfamily S member 1	ILMN_1705403
CYP2U1	cytochrome P450 family 2 subfamily U member 1	ILMN_1790008
CYP3A5	cytochrome P450 family 3 subfamily A member 5	ILMN_1810942
CYP3A7	cytochrome P450 family 3 subfamily A member 7	ILMN_1685043
CYP3A43	cytochrome P450 family 3 subfamily A member 43	ILMN_1673591
CYP4A11	cytochrome P450 family 4 subfamily A member 11	ILMN_1735816
CYP4Z1	cytochrome P450 family 4 subfamily Z member 1	ILMN_1693594

Chaperone and stress responsive proteins

**Symbol**	**Entrez gene name**	**Illumina ID**
CCT7	chaperonin containing TCP1 subunit 7	ILMN_1703718
CLPP	caseinolytic mitochondrial matrix peptidase proteolytic subunit	ILMN_1725705
ERP29	endoplasmic reticulum protein 29	ILMN_1696035
FKBP5	FK506 binding protein 5	ILMN_1778444

HERPUD1	homocysteine inducible ER protein with ubiquitin like domain 1	ILMN_1700346
PPIB	peptidylprolyl isomerase B	ILMN_1711745
PTPLAD1	3-hydroxyacyl-CoA dehydratase 3	ILMN_1658746
STIP1	stress induced phosphoprotein 1	ILMN_1745906
DNAJA1	DnaJ heat shock protein family (Hsp40) member A1	ILMN_1672496
DNAJA2	DnaJ heat shock protein family (Hsp40) member A2	ILMN_1770127
DNAJA3	DnaJ heat shock protein family (Hsp40) member A3	ILMN_1662334
DNAJA4	DnaJ heat shock protein family (Hsp40) member A4	ILMN_1776998
DNAJB1	DnaJ heat shock protein family (Hsp40) member B1	ILMN_1775304
DNAJB11	DnaJ heat shock protein family (Hsp40) member B11	ILMN_1753243
DNAJB12	DnaJ heat shock protein family (Hsp40) member B12	ILMN_1789424
DNAJB13	DnaJ heat shock protein family (Hsp40) member B13	ILMN_1689425
DNAJB14	DnaJ heat shock protein family (Hsp40) member B14	ILMN_1668979
DNAJB2	DnaJ heat shock protein family (Hsp40) member B2	ILMN_1744689
DNAJB4	DnaJ heat shock protein family (Hsp40) member B4	ILMN_1813019
DNAJB5	DnaJ heat shock protein family (Hsp40) member B5	ILMN_1794056
DNAJB6	DnaJ heat shock protein family (Hsp40) member B6	ILMN_1793770
DNAJB7	DnaJ heat shock protein family (Hsp40) member B7	ILMN_1809307
DNAJB8	DnaJ heat shock protein family (Hsp40) member B8	ILMN_1768845
DNAJB9	DnaJ heat shock protein family (Hsp40) member B9	ILMN_1773742
DNAJC1	DnaJ heat shock protein family (Hsp40) member C1	ILMN_1683234
DNAJC10	DnaJ heat shock protein family (Hsp40) member C10	ILMN_1656486
DNAJC11	DnaJ heat shock protein family (Hsp40) member C11	ILMN_1747903
DNAJC13	DnaJ heat shock protein family (Hsp40) member C13	ILMN_1752281
DNAJC14	DnaJ heat shock protein family (Hsp40) member C14	ILMN_1785177
DNAJC15	DnaJ heat shock protein family (Hsp40) member C15	ILMN_1812666
DNAJC16	DnaJ heat shock protein family (Hsp40) member C16	ILMN_1717078
DNAJC17	DnaJ heat shock protein family (Hsp40) member C17	ILMN_1703573
DNAJC18	DnaJ heat shock protein family (Hsp40) member C18	ILMN_1688730
DNAJC19	DnaJ heat shock protein family (Hsp40) member C19	ILMN_1742109
DNAJC21	DnaJ heat shock protein family (Hsp40) member C21	ILMN_1711089
DNAJC3	DnaJ heat shock protein family (Hsp40) member C3	ILMN_1659843
DNAJC4	DnaJ heat shock protein family (Hsp40) member C4	ILMN_1687683
DNAJC5	DnaJ heat shock protein family (Hsp40) member C5	ILMN_1719709
DNAJC5B	DnaJ heat shock protein family (Hsp40) member C5 beta	ILMN_1769403
DNAJC5G	DnaJ heat shock protein family (Hsp40) member C5 gamma	ILMN_1725554
DNAJC7	DnaJ heat shock protein family (Hsp40) member C7	ILMN_1663616
DNAJC8	DnaJ heat shock protein family (Hsp40) member C8	ILMN_1698258
DNAJC9	DnaJ heat shock protein family (Hsp40) member C9	ILMN_1799516
	**Phase III detoxifying proteins**	
**Symbol**	**Entrez gene name**	**Illumina ID**
MRP1	ATP binding cassette subfamily C member 1	ILMN_1802404
MRP2	ATP binding cassette subfamily C member 2	ILMN_1780706
MRP4	ATP binding cassette subfamily C member 4	ILMN_1788457
SR-BI	scavenger receptor class B member 1	ILMN_1668387
	**Ubiquitination and proteasomal degradation**	
**Symbol**	**Entrez gene name**	**Illumina ID**

HIP2	ubiquitin conjugating enzyme E2 K	ILMN_1782954
UB2R1	cell division cycle 34	ILMN_1713006
UBB	ubiquitin B	ILMN_1762436
USP14	ubiquitin specific peptidase 14	ILMN_1806804
VCP	valosin containing protein	ILMN_1665376

NRF2, Nuclear factor erythroid 2-related factor 2

Oxidative stress response genes represented as NRF2 targets were divided into five functional gene sets as indicated based on characterization of the "NRF2-mediated oxidative stress response pathway" by Ingenuity Pathway Analysis. The transcripts are listed with symbol, Entrez gene name and Illumina ID.

Table S2. Quantitative comparison of decidual NRF2 and KEAP1 expression between study groups.

**Comparison**		**Trophoblast density (%)**	**Mean difference**	**Std. Error**	**95% Confidence interval**	***p*-value**
**NRF2 expression**						
PE - FGR	Normal preg	0	-1.95	3.49	(-8.89, 4.99)	0.58
PE + FGR	Normal preg	0	-0.78	3.07	(-6.89, 5.32)	0.80
PE - FGR	PE + FGR	0	-1.17	3.74	(-8.60, 6.27)	0.76
PE - FGR	Normal preg	>0-50	-5.44	3.22	(-11.85, 0.97)	0.10
PE + FGR	Normal preg	>0-50	0,49	2.69	(-4.86, 5.85)	0.86
PE - FGR	PE + FGR	>0-50	-5.93	3.42	(-12.74, 0.87)	0.09
PE - FGR	Normal preg	>50-100	-5.62	3.65	(-12.89, 1.65)	0.13
PE + FGR	Normal preg	>50-100	1.28	3.21	(-5.11, 7.68)	0.69
PE - FGR	PE + FGR	>50-100	-6.90	4.00	(-14.87, 1.06)	0.09
**KEAP1 expression**						
PE - FGR	Normal preg	0	-2.98	2.73	(-8.42, 2.5)	0.28
PE + FGR	Normal preg	0	-0.36	2.48	(-5.29, 4.58)	0.89
PE - FGR	PE + FGR	0	-2.62	2.92	(-8.44, 3.19)	0.37
PE - FGR	Normal preg	>0-50	-2.84	2.64	(-8.09, 2.41)	0.29
PE + FGR	Normal preg	>0-50	1.87	2.29	(-2.68, 6.42)	0.42
PE - FGR	PE + FGR	>0-50	-4.71	2.78	(-10.25, 0.83)	0.09
PE - FGR	Normal preg	>50-100	-5.93	2.96	(-11.84, -0.02)	**0.05**
PE + FGR	Normal preg	>50-100	1.77	2.62	(-3.45, 6.98)	0.50
PE - FGR	PE + FGR	>50-100	-7.69	3.19	(-14.06, -1.33)	**0.02**

FGR, fetal growth restriction; KEAP1, Kelch-like ECH-associated protein 1; NRF2, Nuclear factor erythroid 2-related factor 2; PE, preeclampsia; preg, pregnancy

Quantitative comparison of decidual NRF2- and KEAP1-expression between preeclamptic pregnancies with or without FGR and normal pregnancies. The staining intensity was automatically quantified in MATLAB and compared between the study groups by using a linear regression model (0% trophoblast density) or linear mixed model (>0-50% and >50% trophoblast density). Mean difference for estimated means of staining intensity for decidual NRF2- and KEAP1-expression in the trophoblast density intervals 0%, >0-50% and >50%, comparing normal pregnancies and preeclamptic pregnancies with or without FGR are shown.

As staining intensity is inversely proportional to protein expression, a negative mean difference means that the study group in the left column have a higher protein expression than the study group in the right column and vice versa.

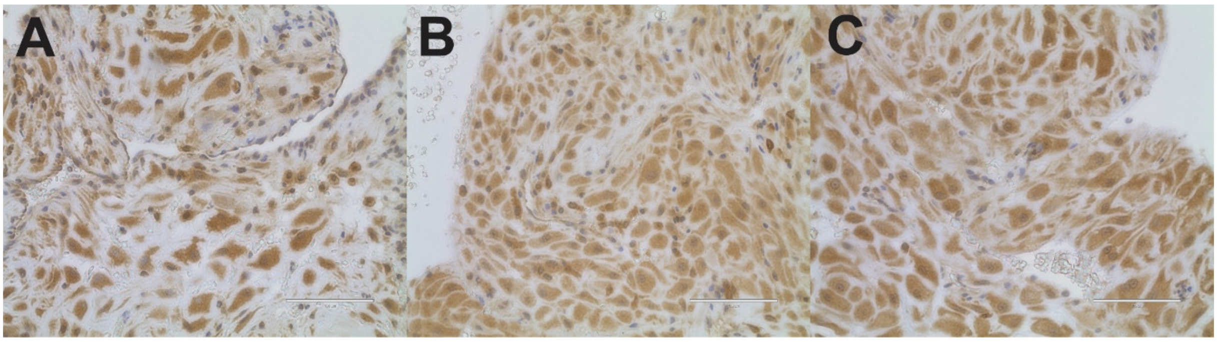

Figure S1. Decidual expression of heme oxygenase 1 (HO-1) in normal pregnancy (A), preeclampsia without fetal growth restriction (B), and preeclampsia with fetal growth restriction (C) (40X magnification). Scale bar 100 μM.

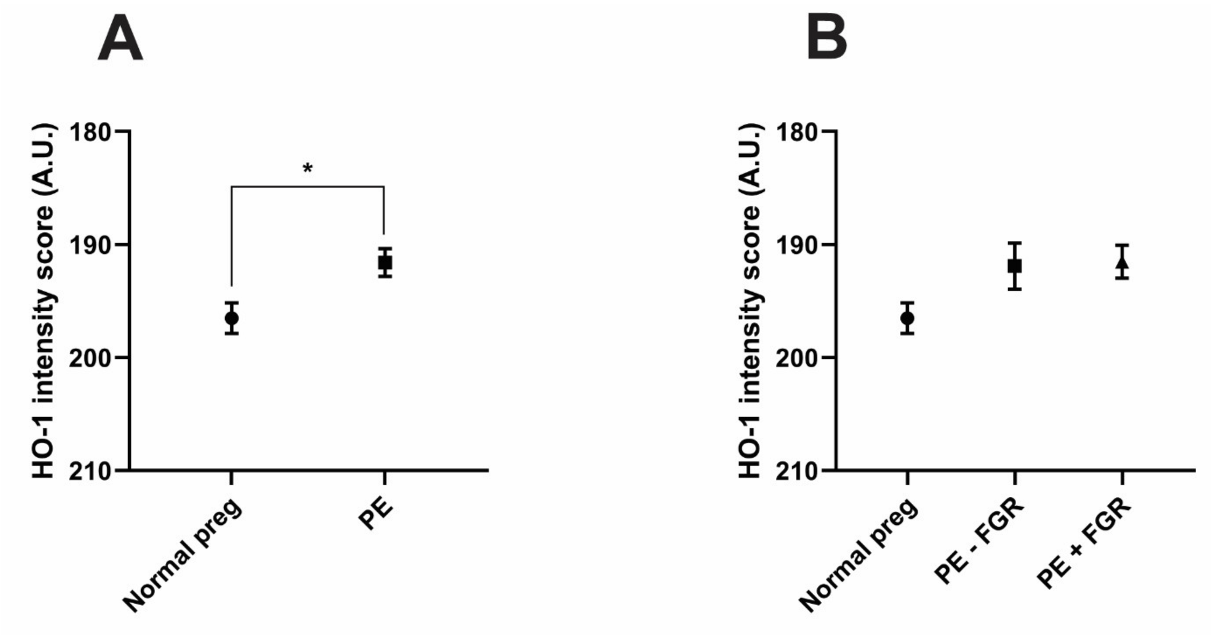

Figure S2. Decidual protein-expression levels of heme oxygenase 1 (HO-1). Expression of HO-1 was compared between normal pregnancies (preg, n = 17) and preeclampsia (PE, n = 16) (A), and between normal pregnancies and PE with (n = 13) or without (n = 3) fetal growth restriction (FGR) (B). Protein-expression levels are shown as estimated mean with standard error of mean. * *$p < 0.05$.*

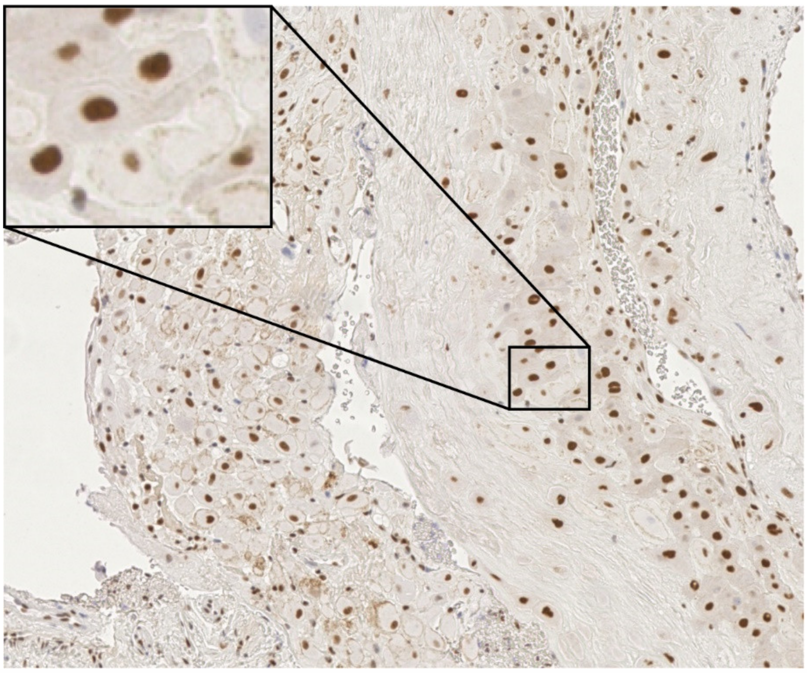

Figure S3. Nuclear expression of Nuclear factor erythroid 2-related factor 2 (NRF2) by staining for phosphorylated NRF2 in decidual tissue from a preeclamptic pregnancy without fetal growth restriction (20X magnification).
